# Supplementary material for: Chemogenetic stimulation of phrenic motor output and diaphragm activity
Source: eLife. 2025 Jun 2;13:RP97846. doi: 10.7554/eLife.97846 (PMC12129449; doi:10.7554/eLife.97846)
Supplement: Supplementary file 1. — Time points are in reference to minutes passed since J60 infusion. Summary data are presented in Figure 1. EMG = electromyography, AUC = area under the curve, RM = repeated measures, df = degrees of freedom. Bolded p-values indicate p < 0.05. [file elife-97846-supp1.docx]

| Main Effects | | | | | |
| --- | --- | --- | --- | --- | --- |
| Outcome | Hemi-diaphragm | Test | df | Test statistic | p value |
| Diaphragm EMG AUC | left | One-way RM ANOVA | 6,68 | F = 4.008 | **0.002** |
|  | right | One-way RM ANOVA | 6,68 | F = 4.054 | **0.002** |
| Diaphragm EMG peak-to-peak amplitude | left | One-way RM ANOVA | 6,68 | F = 2.213 | 0.056 |
|  | right | One-way RM ANOVA | 6,68 | F = 3.191 | **0.01** |
| Diaphragm EMG tonic activity | left | One-way RM ANOVA | 6,68 | F = 2.256 | 0.052 |
|  | right | One-way RM ANOVA | 6,68 | F = 5.225 | **< 0.001** |
| Respiratory Rate | NA | One-way RM ANOVA | 6,68 | F = 0.419 | 0.863 |
| Post-Hoc Tests (Tukey Test) | | | | | |
| Outcome | Hemi-diaphragm | Comparison | Diff of Ranks | q | p |
| Diaphragm EMG AUC | left | 15 min vs. Saline | 60.488 | 4.972 | **0.015** |
|  |  | 60 min vs. Saline | 68.183 | 5.064 | **0.013** |
|  |  | 90 min vs. Saline | 66.079 | 4.446 | **0.041** |
|  |  |  |  |  |  |
| Diaphragm EMG AUC | right | 15 min vs. Saline | 41.481 | 4.41 | **0.044** |
|  |  | 60 min vs. Saline | 54.104 | 5.197 | **0.01** |
|  |  | 90 min vs. Saline | 51.214 | 4.457 | **0.04** |
|  |  |  |  |  |  |
| Diaphragm EMG peak-to-peak amplitude | right | 90 min vs. Saline | 40.925 | 4.947 | **0.016** |
|  |  |  |  |  |  |
| Diaphragm EMG tonic activity | right | 15 min vs. Baseline | 124.737 | 5.044 | **0.013** |
|  |  | 15 min vs. Saline | 135.783 | 5.49 | **0.005** |
|  |  | 30 min vs. Baseline | 110.583 | 4.471 | **0.039** |
|  |  | 30 min vs. Saline | 121.628 | 4.918 | **0.017** |
|  |  | 90 min vs. Baseline | 140.208 | 4.641 | **0.029** |
|  |  | 90 min vs. Saline | 151.254 | 5.006 | **0.014** |

**Supplementary File 1. *Statistical summary for the impact of DREADD activation on diaphragm EMG in wild-type mice.*** Time points are in reference to minutes passed since J60 infusion. Summary data is presented in Figure 1. EMG = electromyography, AUC = area under the curve, RM = repeated measures, df = degrees of freedom. Bolded p-values indicate p < 0.05.
