# Supplementary material for: Chemogenetic stimulation of phrenic motor output and diaphragm activity
Source: eLife. 2025 Jun 2;13:RP97846. doi: 10.7554/eLife.97846 (PMC12129449; doi:10.7554/eLife.97846)
Supplement: Supplementary file 2. — Time points are in reference to minutes passed since J60 infusion. Summary data are presented in Figure 2. EMG = electromyography, AUC = area under the curve, RM = repeated measures, df = degrees of freedom. Bolded p-values indicate p < 0.05. [file elife-97846-supp2.docx]

| Main Effects | | | | | |
| --- | --- | --- | --- | --- | --- |
| Outcome | Hemi-diaphragm | Test | df | Test statistic | p value |
| Diaphragm EMG AUC | left | Friedman RM analysis on ranks | 6 | Chi-square = 16.619 | **0.011** |
|  | right | Friedman RM analysis on ranks | 6 | Chi-square = 40.143 | **< 0.001** |
| Diaphragm EMG peak-to-peak amplitude | left | One-way RM ANOVA | 6,62 | F = 3.05 | **0.013** |
|  | right | Friedman RM analysis on ranks | 6 | Chi-square = 36.667 | **< 0.001** |
| Diaphragm EMG tonic activity | left | Friedman RM analysis on ranks | 6 | Chi-square = 20.952 | **0.002** |
|  | right | One-way RM ANOVA | 6,62 | F = 10.076 | **< 0.001** |
| Respiratory Rate | NA | One-way RM ANOVA | 6,62 | F = 25.13 | **< 0.001** |
| Post-Hoc Tests (Tukey Test) | | | | | |
| Outcome | Hemi-diaphragm | Comparison | Diff of Ranks | q | p |
| Diaphragm EMG AUC | left | 30 min vs. Saline | 32 | 4.938 | **0.009** |
|  |  |  |  |  |  |
| Diaphragm EMG AUC | right | 15 min vs. Baseline | 31 | 4.783 | **0.013** |
|  |  | 15 min vs. Saline | 38 | 5.864 | **< 0.001** |
|  |  | 30 min vs. Baseline | 35 | 5.401 | **0.003** |
|  |  | 30 min vs. Saline | 42 | 6.481 | **< 0.001** |
|  |  | 60 min vs. Baseline | 32 | 4.938 | **0.009** |
|  |  | 60 min vs. Saline | 39 | 6.018 | **< 0.001** |
|  |  | 90 min vs. Baseline | 27 | 4.166 | **0.05** |
|  |  | 90 min vs. Saline | 34 | 5.246 | **0.004** |
|  |  |  |  |  |  |
| Diaphragm EMG peak-to-peak amplitude | left | 30 min vs. Saline | 32.824 | 4.447 | **0.042** |
|  |  |  |  |  |  |
| Diaphragm EMG peak-to-peak amplitude | right | 15 min vs. Baseline | 31 | 4.783 | **0.013** |
|  |  | 15 min vs. Saline | 36 | 5.555 | **0.002** |
|  |  | 30 min vs. Baseline | 34 | 5.246 | **0.004** |
|  |  | 30 min vs. Saline | 39 | 6.018 | **< 0.001** |
|  |  | 60 min vs. Baseline | 32 | 4.938 | **0.009** |
|  |  | 60 min vs. Saline | 37 | 5.709 | **0.001** |
|  |  | 90 min vs. Saline | 31 | 4.783 | **0.013** |
|  |  |  |  |  |  |
| Diaphragm EMG tonic activity | left | 5 min vs. Baseline | 28 | 4.32 | **0.037** |
|  |  | 15 min vs. Baseline | 29 | 4.475 | **0.026** |
|  |  | 90 min vs. Baseline | 35 | 5.401 | **0.003** |
|  |  |  |  |  |  |
| Diaphragm EMG tonic activity | right | 5 min vs. Baseline | 295.193 | 5.582 | **0.005** |
|  |  | 5 min vs. Saline | 297.174 | 5.619 | **0.004** |
|  |  | 15 min vs. Baseline | 375.713 | 7.104 | **< 0.001** |
|  |  | 15 min vs. Saline | 377.694 | 7.142 | **< 0.001** |

Supplementary File 2. Continued.

| Outcome | Hemi-diaphragm | Comparison | Diff of Ranks | q | p |
| --- | --- | --- | --- | --- | --- |
| Diaphragm EMG tonic activity | right | 30 min vs. Baseline | 347.212 | 6.565 | **< 0.001** |
|  |  | 30 min vs. Saline | 349.193 | 6.603 | **< 0.001** |
|  |  | 60 min vs. Baseline | 317.232 | 5.998 | **0.002** |
|  |  | 60 min vs. Saline | 319.213 | 6.036 | **0.002** |
|  |  | 90 min vs. Baseline | 358.443 | 6.778 | **< 0.001** |
|  |  | 90 min vs. Saline | 360.424 | 6.815 | **< 0.001** |
|  |  |  |  |  |  |
| Respiratory Rate | NA | 5 min vs. 30 min | 5.296 | 4.733 | **0.025** |
|  |  | 5 min vs. 60 min | 7.296 | 6.52 | **< 0.001** |
|  |  | 5 min vs. 90 min | 11.889 | 10.624 | **< 0.001** |
|  |  | 15 min vs. Baseline | 5.778 | 5.163 | **0.011** |
|  |  | 15 min vs. Saline | 5.963 | 5.328 | **0.008** |
|  |  | 15 min vs. 90 min | 9.222 | 8.241 | **< 0.001** |
|  |  | 30 min vs. Baseline | 8.407 | 7.513 | **< 0.001** |
|  |  | 30 min vs. Saline | 8.593 | 7.678 | **< 0.001** |
|  |  | 30 min vs. 90 min | 6.593 | 5.891 | **0.002** |
|  |  | 60 min vs. Baseline | 10.407 | 9.3 | **< 0.001** |
|  |  | 60 min vs. Saline | 10.593 | 9.465 | **< 0.001** |
|  |  | 90 min vs. Baseline | 15 | 13.404 | **< 0.001** |
|  |  | 90 min vs. Saline | 15.185 | 13.569 | **< 0.001** |

**Supplementary File 2. *Statistical summary for the impact of DREADD activation on diaphragm EMG in ChAT-Cre mice.*** Time points are in reference to minutes passed since J60 infusion. Summary data are presented in Figure 2. EMG = electromyography, AUC = area under the curve, RM = repeated measures, df = degrees of freedom. Bolded p-values indicate p < 0.05.
