# Supplementary material for: Chemogenetic stimulation of phrenic motor output and diaphragm activity
Source: eLife. 2025 Jun 2;13:RP97846. doi: 10.7554/eLife.97846 (PMC12129449; doi:10.7554/eLife.97846)
Supplement: Supplementary file 3. — Summary data are presented in Figure 3. EMG = electromyography, AUC = area under the curve, df = degrees of freedom. Bolded p-values indicate p < 0.05. [file elife-97846-supp3.docx]

| Outcome | Hemi-diaphragm | Test | df | Test statistic | p value |
| --- | --- | --- | --- | --- | --- |
| Diaphragm EMG AUC | left | Student's t-test | 18 | t = 0.00310 | 0.998 |
|  | right | Student's t-test | 18 | t = -2.913 | **0.0417** |
|  |  |  |  |  |  |
| Diaphragm EMG peak-to-peak amplitude | left | Student's t-test | 18 | t = -0.295 | 0.771 |
|  | right | Student's t-test | 18 | t = -3.294 | **0.00403** |
|  |  |  |  |  |  |
| Diaphragm EMG tonic activity | left | Mann-Whitney Rank Sum test | 18 | Mann-Whitney U Statistic = 31.000 | 0.160 |
|  | right | Student's t-test | 18 | t = -3.596 | **0.00207** |
|  |  |  |  |  |  |
| Respiratory Rate | NA | Mann-Whitney Rank Sum test | 18 | Mann-Whitney U Statistic = 38.000 | 0.382 |

**Supplementary File 3. *Statistical summary for the impact of DREADD activation on diaphragm EMG in wild-type mice vs. ChAT-Cre mice at the 30-min post-J60 infusion time point.*** Summary data are presented in Figure 3. EMG = electromyography, AUC = area under the curve, df = degrees of freedom. Bolded p-values indicate p < 0.05.
