# Supplementary material for: Chemogenetic stimulation of phrenic motor output and diaphragm activity
Source: eLife. 2025 Jun 2;13:RP97846. doi: 10.7554/eLife.97846 (PMC12129449; doi:10.7554/eLife.97846)
Supplement: Supplementary file 4. — Each outcome measure is presented normalized to body weight (with the exception of respiratory rate) and normalized to values at baseline. Summary data are presented in Figure 5. RM = repeated measures, ml/kg = milliliters of air per kilogram of animal’s body weight, df = degrees of freedom. Bolded p-values indicate p < 0.05. [file elife-97846-supp4.docx]

| Outcome | Test | Normalization | Main effects | df | Test statistic | p value |
| --- | --- | --- | --- | --- | --- | --- |
| Tidal Volume | Two-way RM ANOVA | Normalized to body weight (ml/kg) | Treatment | 1,89 | F = 6.286 | **0.037** |
|  |  |  | Time | 4,89 | F = 9.817 | **< 0.001** |
|  |  |  | Interaction | 4,89 | F = 0.969 | 0.438 |
|  |  |  |  |  |  |  |
|  |  | Normalized to baseline | Treatment | 1,89 | F = 3.7 | 0.091 |
|  |  |  | Time | 4,89 | F = 9.448 | **< 0.001** |
|  |  |  | Interaction | 4,89 | F = 1.049 | 0.398 |
|  |  |  |  |  |  |  |
|  |  |  |  |  |  |  |
| Respiratory Rate | Two-way RM ANOVA | Raw values | Treatment | 1,89 | F = 0.329 | 0.582 |
|  |  |  | Time | 4,89 | F = 14.326 | **< 0.001** |
|  |  |  | Interaction | 4,89 | F = 1.640 | 0.188 |
|  |  |  |  |  |  |  |
|  |  | Normalized to baseline | Treatment | 1,89 | F = 0.0885 | 0.774 |
|  |  |  | Time | 4,89 | F = 11.862 | **< 0.001** |
|  |  |  | Interaction | 4,89 | F = 1.310 | 0.287 |
|  |  |  |  |  |  |  |
|  |  |  |  |  |  |  |
| Minute Ventilation | Two-way RM ANOVA | Normalized to body weight (ml/kg) | Treatment | 1,89 | F = 2.011 | 0.194 |
|  |  |  | Time | 4,89 | F = 12.875 | **< 0.001** |
|  |  |  | Interaction | 4,89 | F = 1.215 | 0.324 |
|  |  |  |  |  |  |  |
|  |  | Normalized to baseline | Treatment | 1,89 | F = 1.043 | 0.337 |
|  |  |  | Time | 4,89 | F = 10.776 | **< 0.001** |
|  |  |  | Interaction | 4,89 | F = 0.490 | 0.49 |

**Supplementary File 4. *Statistical summary for the impact of DREADD activation on plethysmography outcomes in unanesthetized ChAT-Cre rats using two-way repeated measures ANOVAs.*** Each outcome measure is presented normalized to body weight (with the exception of respiratory rate) and normalized to values at baseline. Summary data are presented in Figure 5. RM = repeated measures, ml/kg = milliliters of air per kilogram of animal’s body weight, df = degrees of freedom. Bolded p-values indicate p < 0.05.
