# Supplementary material for: Chemogenetic stimulation of phrenic motor output and diaphragm activity
Source: eLife. 2025 Jun 2;13:RP97846. doi: 10.7554/eLife.97846 (PMC12129449; doi:10.7554/eLife.97846)
Supplement: Supplementary file 5. — Time points are in reference to minutes passed since J60 infusion. Summary data are presented in Figure 6. RM = repeated measures, df = degrees of freedom. Bolded p-values indicate p < 0.05. [file elife-97846-supp5.docx]

| Main Effects | | | | | |
| --- | --- | --- | --- | --- | --- |
| Outcome | Nerve | Test | df | Test statistic | p value |
| Phrenic peak-to-peak amplitude (normalized to baseline) | left | One-way RM ANOVA | 6,62 | F = 13.355 | **< 0.001** |
|  | right | Friedman Repeated Measures Analysis of Variance on Ranks | 6 | Chi-square = 35.619 | **< 0.001** |
|  |  |  |  |  |  |
| Phrenic tonic activity (normalized to baseline) | left | Friedman Repeated Measures Analysis of Variance on Ranks | 6 | Chi-square = 36.714 | **< 0.001** |
|  | right | One-way RM ANOVA | 6,62 | F = 14.544 | **< 0.001** |
|  |  |  |  |  |  |
| Heart rate | NA | Friedman Repeated Measures Analysis of Variance on Ranks | 6 | Chi-square = 4.667 | 0.587 |
| Systolic blood pressure | NA | One-way RM ANOVA | 6,62 | F = 5.403 | **< 0.001** |
| Diastolic blood pressure | NA | One-way RM ANOVA | 6,62 | F = 12.460 | **< 0.001** |
| Mean arterial blood pressure | NA | One-way RM ANOVA | 6,62 | F = 9.422 | **< 0.001** |
| Respiratory Rate | NA | One-way RM ANOVA | 6,62 | F = 1.285 | 0.282 |
| Post-Hoc Tests (Tukey Test) | | | | | |
| Outcome | Nerve | Comparison | Diff of Ranks | q | p |
| Phrenic peak-to-peak amplitude (normalized to baseline) | left | 5 min vs. Baseline | 111.026 | 6.52 | **< 0.001** |
|  |  | 5 min vs. Saline | 86.692 | 5.091 | **0.013** |
|  |  | 15 min vs. Baseline | 133.337 | 7.83 | **< 0.001** |
|  |  | 15 min vs. Saline | 109.003 | 6.401 | **< 0.001** |
|  |  | 30 min vs. Baseline | 152.488 | 8.955 | **< 0.001** |
|  |  | 30 min vs. Saline | 128.153 | 7.526 | **< 0.001** |
|  |  | 60 min vs. Baseline | 140.658 | 8.26 | **< 0.001** |
|  |  | 60 min vs. Saline | 116.324 | 6.831 | **< 0.001** |
|  |  | 90 min vs. Baseline | 143.291 | 8.415 | **< 0.001** |
|  |  | 90 min vs. Saline | 118.956 | 6.986 | **< 0.001** |
|  |  |  |  |  |  |
|  | right | 15 min vs. Baseline | 32 | 4.938 | **0.009** |
|  |  | 15 min vs. Saline | 29 | 4.475 | **0.026** |
|  |  | 30 min vs. Baseline | 37 | 5.709 | **0.001** |
|  |  | 30 min vs. Saline | 34 | 5.246 | **0.004** |
|  |  | 60 min vs. Baseline | 35 | 5.401 | **0.003** |
|  |  | 60 min vs. Saline | 32 | 4.938 | **0.009** |
|  |  | 90 min vs. Baseline | 35 | 5.401 | **0.003** |
|  |  | 90 min vs. Saline | 32 | 4.938 | **0.009** |
|  |  |  |  |  |  |
| Phrenic tonic activity (normalized to baseline) | left | 30 min vs. Baseline | 32 | 4.938 | **0.009** |
|  |  | 30 min vs. Saline | 42 | 6.481 | **<0.001** |
|  |  | 60 min vs. Baseline | 33 | 5.092 | **0.006** |
|  |  | 60 min vs. Saline | 43 | 6.635 | **<0.001** |
|  |  | 90 min vs. Saline | 31 | 4.783 | **0.013** |

Supplementary File 5. Continued.

| Outcome | Nerve | Comparison | Diff of Ranks | q | p |
| --- | --- | --- | --- | --- | --- |
| Phrenic tonic activity (normalized to baseline) | right | 15 min vs. Baseline | 161.81 | 6.077 | **0.002** |
|  |  | 15 min vs. Saline | 167.132 | 6.277 | **0.001** |
|  |  | 30 min vs. Baseline | 227.376 | 8.54 | **< 0.001** |
|  |  | 30 min vs. Saline | 232.698 | 8.74 | **< 0.001** |
|  |  | 30 min vs. 5 min | 151.316 | 5.683 | **0.004** |
|  |  | 60 min vs. Baseline | 222.864 | 8.37 | **< 0.001** |
|  |  | 60 min vs. Saline | 228.186 | 8.57 | **< 0.001** |
|  |  | 60 min vs. 5 min | 146.805 | 5.514 | **0.005** |
|  |  | 90 min vs. Baseline | 198.832 | 7.468 | **< 0.001** |
|  |  | 90 min vs. Saline | 204.154 | 7.668 | **< 0.001** |
|  |  | 90 min vs. 5 min | 122.773 | 4.611 | **0.031** |
|  |  |  |  |  |  |
| Systolic blood pressure | NA | 5 min vs. Baseline | 33.737 | 6.017 | **0.002** |
|  |  | 5 min vs. Saline | 33.977 | 6.06 | **0.002** |
|  |  | 15 min vs. Baseline | 26.184 | 4.67 | **0.028** |
|  |  | 15 min vs. Saline | 26.423 | 4.713 | **0.026** |
|  |  | 90 min vs. Baseline | 24.843 | 4.431 | **0.043** |
|  |  | 90 min vs. Saline | 25.083 | 4.474 | **0.04** |
|  |  |  |  |  |  |
| Diastolic blood pressure | NA | 5 min vs. Baseline | 27.201 | 9.186 | **< 0.001** |
|  |  | 5 min vs. Saline | 20.438 | 6.902 | **< 0.001** |
|  |  | 15 min vs. Baseline | 23.277 | 7.861 | **< 0.001** |
|  |  | 15 min vs. Saline | 16.515 | 5.577 | **0.005** |
|  |  | 30 min vs. Baseline | 17.364 | 5.864 | **0.003** |
|  |  | 60 min vs. Baseline | 21.693 | 7.326 | **< 0.001** |
|  |  | 60 min vs. Saline | 14.931 | 5.042 | **0.014** |
|  |  | 90 min vs. Baseline | 26.929 | 9.094 | **< 0.001** |
|  |  | 90 min vs. Saline | 20.167 | 6.811 | **< 0.001** |
|  |  |  |  |  |  |
| Mean arterial blood pressure | NA | 5 min vs. Baseline | 29.379 | 8.053 | **< 0.001** |
|  |  | 5 min vs. Saline | 24.951 | 6.839 | **< 0.001** |
|  |  | 15 min vs. Baseline | 24.246 | 6.646 | **< 0.001** |
|  |  | 15 min vs. Saline | 19.818 | 5.432 | **0.006** |
|  |  | 30 min vs. Baseline | 17.025 | 4.667 | **0.028** |
|  |  | 60 min vs. Baseline | 20.993 | 5.754 | **0.003** |
|  |  | 60 min vs. Saline | 16.565 | 4.54 | **0.036** |
|  |  | 90 min vs. Baseline | 26.234 | 7.191 | **< 0.001** |
|  |  | 90 min vs. Saline | 21.806 | 5.977 | **0.002** |

**Supplementary File 5. *Statistical summary for the impact of DREADD activation on phrenic nerve activity in ChAT-Cre rats.*** Time points are in reference to minutes passed since J60 infusion. Summary data are presented in Figure 6. RM = repeated measures, df = degrees of freedom. Bolded p-values indicate p < 0.05.
